# Racial and Ethnic Disparities in Access to Home‐Based Renal Replacement Therapies Among Incident End‐Stage Renal Disease Patients in the United States

**DOI:** 10.1111/sdi.70050

**Published:** 2026-10-04

**Authors:** Andres Mauricio Garcia‐Sierra, Bernardo Ramírez, Marcia Tan

**Affiliations:** ^1^ Department of Public Health Sciences University of Chicago Chicago Illinois USA; ^2^ School of Global Health and Informatics University of Central Florida Orlando Florida USA

**Keywords:** dialysis, End‐stage renal disease, health equity, racial disparities, USRDS

## Abstract

**Background:**

Racial and ethnic disparities in access to home‐based renal replacement therapies (RRT) remain a major concern in the management of end‐stage renal disease (ESRD) in the United States. Despite national efforts to expand home hemodialysis (HHD) and peritoneal dialysis (PD), inequities at treatment initiation persist. Therefore, the goal was to evaluate racial and ethnic disparities in the initial modality of RRT among incident adult ESRD patients in the United Stated from 2014 to 2020.

**Methods:**

This retrospective, pooled cross‐sectional study used United States Renal Data System (USRDS) data on adults initiating dialysis between 2014 and 2020. Descriptive analyses compared biological, environmental, and behavioral determinants across modalities. Poisson regression models with robust variance estimated adjusted incidence rate ratios (IRRs) for initiating HHD or PD versus in‐center hemodialysis (ICHD), controlling for demographic, clinical, socioeconomic, and behavioral factors within an integrated health risk management framework.

**Results:**

Among 3.49 million incident ESRD cases, 82.8% initiated ICHD, 15.5% PD, and 1.7% HHD. Significant disparities were observed by race/ethnicity. Compared with White patients, Black patients had markedly lower adjusted incidence rates of initiating HHD (IRR 0.69; 95% CI 0.67–0.72) and PD (IRR 0.55; 95% CI 0.55–0.56). Hispanic or Latino patients similarly demonstrated reduced initiation of HHD (IRR 0.42; 95% CI 0.40–0.45) and PD (IRR 0.64; 95% CI 0.64–0.65). Asian patients had a modestly higher adjusted rate of PD initiation (IRR 1.08; 95% CI 1.06–1.09) but lower rates of HHD. Other racial groups also showed consistently reduced use of home modalities.

**Conclusions:**

Substantial racial and ethnic disparities persist in the initiation of home dialysis in the U.S. While structural and system‐level factors likely contribute, patient‐level clinical considerations, cultural norms, social support availability, and provider practice patterns also play important roles.

## 1 Introduction

End‐stage renal disease (ESRD) represents the final stage of chronic kidney disease (CKD), requiring renal replacement therapy (RRT) or transplantation to sustain life [1]. In the United States, over 800,000 individuals live with ESRD, with the majority undergoing dialysis treatment [2]. At initiation, patients typically receive one of three RRT modalities: in‐center hemodialysis (ICHD), home hemodialysis (HHD), or peritoneal dialysis (PD). Although these modalities are clinically viable, they vary in terms of patient autonomy, cost, and accessibility.

Disparities in the utilization of RRTs, especially at the initiation stage, have long been documented across racial and ethnic groups. Studies consistently show that Black and Hispanic patients are more likely to begin with ICHD and less likely to initiate PD or HHD compared to White patients, even after controlling for clinical and demographic variables [3, 4]. These disparities may reflect structural inequities, implicit provider bias, language barriers, and differences in access to pre‐dialysis care or patient education [5, 6].

This study uses the Integrated Health Risk Management (IHRM) framework as an organizing structure to examine factors associated with disparities in renal replacement therapy (RRT) initiation. Originally conceptualized by Krewski et al. (2007), the IHRM framework integrates health risk science and population health principles by considering how biological, environmental, and behavioral determinants interact to influence health outcomes [7]. Although other frameworks, such as the National Institute on Minority Health and Health Disparities (NIMHD) Research Framework, are more commonly applied in health disparities research, the IHRM model was selected because it provides a multidimensional structure that aligns with the study objective of evaluating factors associated with dialysis modality selection at ESRD onset.

Building on this framework, a risk management conceptual model was developed for this study to analyze RRT selection among ESRD patients (Figure 1). The model classifies determinants into three domains: biological (e.g., age, sex, and comorbidities), environmental (e.g., employment status and insurance coverage), and behavioral (e.g., substance use and functional status). This approach provides a structured framework for examining how individual characteristics and contextual factors are associated with the likelihood of initiating specific dialysis modalities, with particular attention to differences across racial and ethnic groups.

**FIGURE 1:**
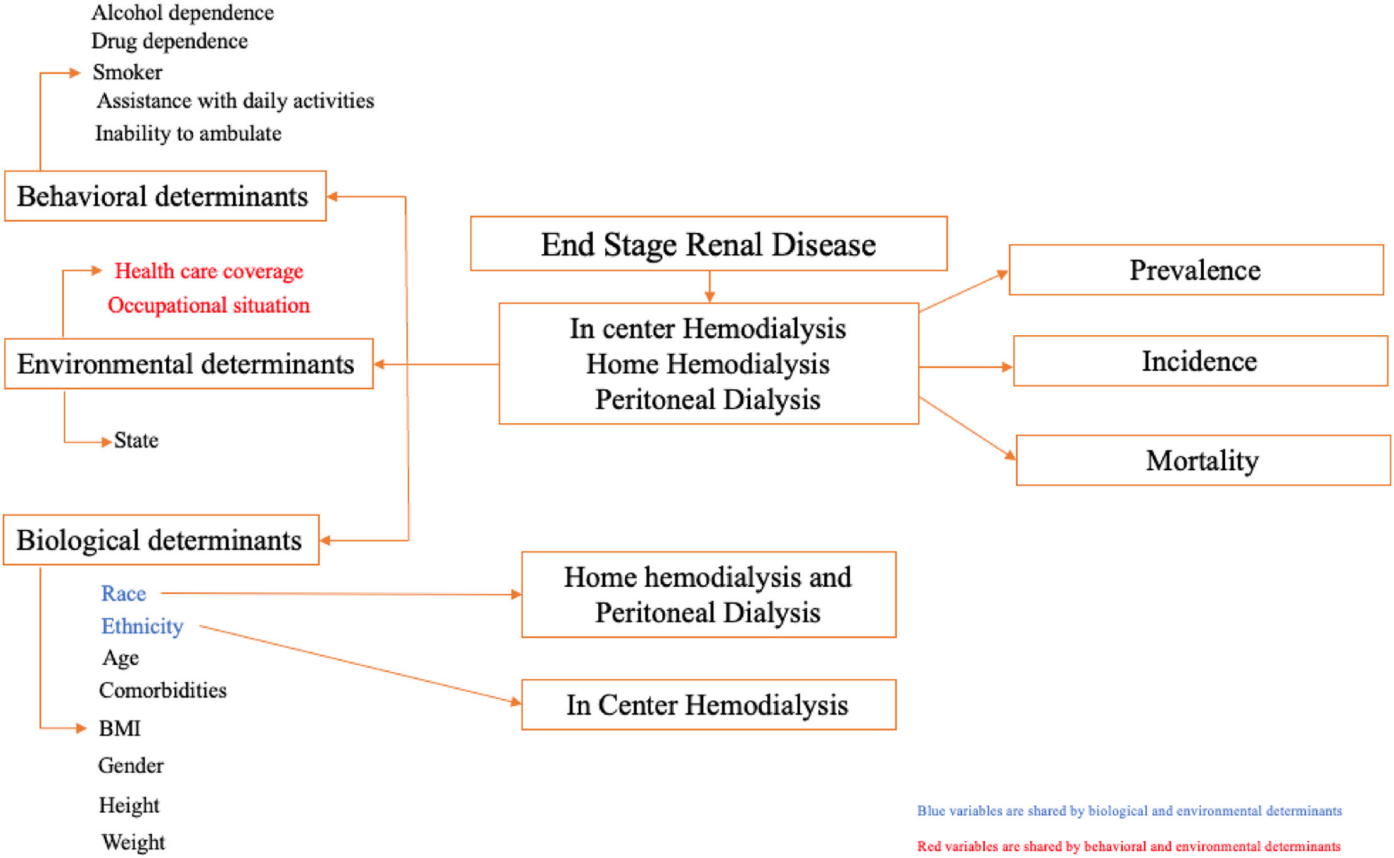
A risk management conceptual model. *Source:* Authors' own elaboration.

Federal initiatives, such as the *Advancing American Kidney Health* strategy, have prioritized increasing the use of home‐based therapies [8]. However, achieving equity in access remains a challenge. While prior research has examined modality use broadly, fewer studies have explored how racial and ethnic disparities manifest at the critical point of initiation: a moment that can influence long‐term treatment patterns and outcomes.

To address this gap, the present study analyzes national‐level data from the USRDS (2014–2020) to investigate racial and ethnic disparities in the initial dialysis modality among incident ESRD cases in the United States. The goal is to identify whether inequities persist in the initial treatment decisions and to inform targeted strategies that promote equitable access to PD and HHD for all patients.

## 2 Methods

### 2.1 Study Design and Data Source

This study employed a retrospective, pooled cross‐sectional design to evaluate racial and ethnic disparities in the initial modality of renal replacement therapy (RRT) among incident end‐stage renal disease (ESRD) patients in the United States between 2014 and 2020. Data were drawn from the United States Renal Data System (USRDS), a comprehensive national registry managed by the National Institutes of Health. The analysis utilized patient‐level information from the Medical Evidence Form (CMS‐2728) and the Treatment History Files, which together provided detailed data on demographic characteristics, comorbidities, and the initial dialysis modality. The determination of dialysis modality switches followed the 60‐day rule used by the USRDS whereby any change in modality lasting less than 60 days was not recorded as a modality switch [2].

### 2.2 Study Population

The study population included all adult individuals aged 18 years or older who initiated chronic dialysis between January 1, 2014, and December 31, 2020. Patients were eligible for inclusion if they had complete information on race/ethnicity and dialysis modality at initiation. Individuals were excluded if they began treatment with kidney transplantation, lacked documentation of initial dialysis modality, or had missing data on race/ethnicity. To ensure the accuracy of modality classification, only patients who remained on the same dialysis modality for at least 60 consecutive days after initiation were retained for analysis. To address missing data, excluded individuals with missing race/ethnicity values were excluded, as these were essential to defining the exposure categories. No imputation methods were applied. This approach aligns with standard practice for large administrative datasets and is consistent with prior studies using USRDS data [2].

The derivation of the analytic cohort is shown in Figure 2. The patients file initially contained 3,254,323 observations. We excluded individuals who died before the modality ascertainment window (*n* = 771,644), whose first recorded treatment was kidney transplantation (*n* = 74,712) or acute dialysis (*n* = 8159), those younger than 18 years (*n* = 25,346), and cases with missing race/ethnicity data (*n* = 994), yielding 2,373,468 individuals.

**FIGURE 2:**
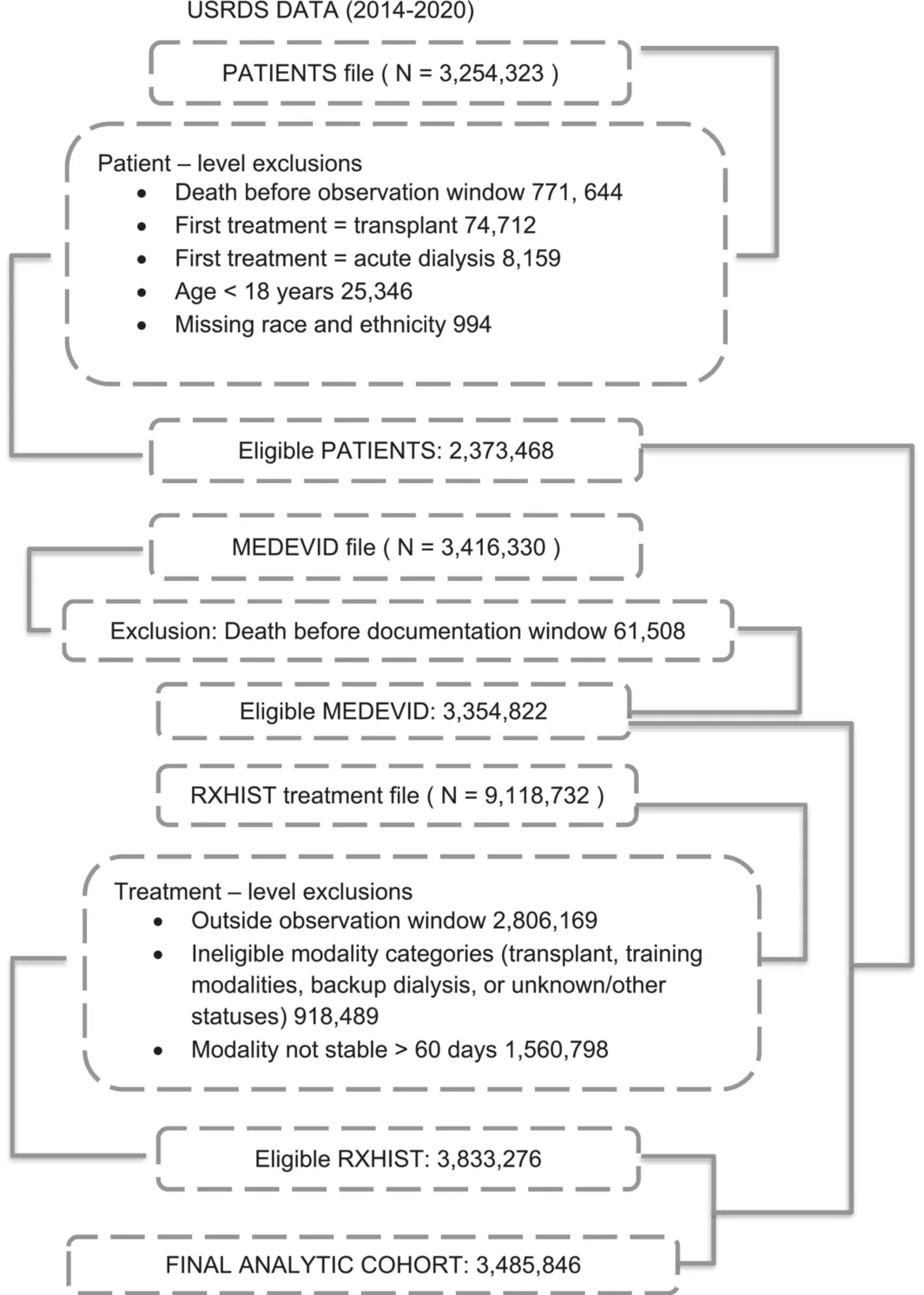
Flowchart of cohort construction and application of inclusion and exclusion criteria. USRDS DATA (2014–2020).

The MEDEVID file contained 3,416,330 records; individuals who died prior to the observation period were excluded (*n* = 61,508), resulting in 3,354,822 records available for merging.

The RXHIST dataset contained 9,118,732 treatment records. We excluded treatments occurring before (*n* = 2,805,161) or after (*n* = 1008) the observation window, records belonging to excluded modality categories (*n* = 918,489), and individuals whose modality was not stable for at least 60 days (*n* = 1,560,798), leaving 3,833,276 observations eligible for modality assignment.

After merging patient, modality, and medical evidence files, unmatched records were removed (85,391 from PATIENTS and 249,388 from RXHIST). The final analytic sample consisted of 3,485,846 adult incident ESRD patients with confirmed dialysis modality at day 60.

### 2.3 Exposure and Outcome Variables

The primary exposure variables were race/ethnicity. Race and ethnicity were recategorized as a race/ethnicity variable like NHANES does [9]: White, Black or African American, Asian, American Indian or Alaska Native, Native Hawaiian or Pacific Islander, Other, and Hispanic or Latino.

The primary outcome was initial renal replacement therapy (RRT) modality, categorized as in‐center hemodialysis (ICHD), home hemodialysis (HHD), or peritoneal dialysis (PD). Patients were required to remain on the initial modality for ≥ 60 days, consistent with USRDS convention for establishing modality stability. In regression analyses, ICHD served as the reference category.

### 2.4 Covariates

Covariates were selected based on the Integrated Health Risk Management (IHRM) framework and prior evidence linking biological, environmental, and behavioral factors to dialysis modality selection (Table S1). Age, measured continuously at the time of ESRD onset, and gender, recorded as male or female, were included. Body mass index (BMI) was calculated from height and weight reported on Form CMS‐2728. The primary cause of ESRD, categorized as diabetes, hypertension, glomerulonephritis, cystic kidney disease, or other etiologies, was derived from CMS‐2728 checkboxes.

Comorbid conditions documented on CMS‐2728, including diabetes, hypertension, COPD, cancer, AIDS, glomerulonephritis, and cystic kidney disease, were treated as binary variables. Environmental variables included employment status, categorized as full‐time employed, part‐time employed, unemployed, homemaker, retired due to age, or retired due to disability. Health care coverage at ESRD onset was classified as Medicare, Medicaid, Department of Veterans Affairs (DVA), other insurance, or uninsured. In addition, access to specialty kidney care prior to ESRD onset was assessed using duration of pre‐ESKD nephrology care, derived from the CMS Medical Evidence Form (CMS‐2728). This variable was categorized as no nephrology care, < 6 months, 6–12 months, or > 12 months of nephrology care before ESRD onset and was included as an environmental determinant reflecting access to specialty care and opportunities for predialysis education and preparation.

Behavioral and functional characteristics, also derived from CMS‐2728, included smoking, alcohol dependence, drug dependence, need for assistance with activities of daily living (ADLs), and inability to ambulate and were treated as binary indicators. No imputation procedures were used.

### 2.5 Statistical Analysis

Descriptive statistics were first generated to characterize the study population by initial dialysis modality (ICHD, HHD, PD) and by race/ethnicity. Categorical variables were compared using chi‐square tests, and continuous variables were assessed using one‐way ANOVA or the Kruskal–Wallis test, depending on the distributional characteristics of each variable. Race and Hispanic ethnicity were combined into a single mutually exclusive race/ethnicity variable. To evaluate the association between race/ethnicity and the likelihood of initiating home hemodialysis (HHD) or peritoneal dialysis (PD), Poisson regression models with robust standard errors were estimated. This analytic approach was selected because the prevalence of PD (approximately 15.5%) and HHD (approximately 1.7%) in the cohort is sufficiently common that logistic regression odds ratios could meaningfully overestimate effect sizes. Poisson regression with robust variance yields incidence rate ratios (IRRs), which provide more interpretable and numerically stable estimates of relative risk for binary outcomes in this context.

For each modality outcome, HHD versus ICHD and PD versus ICHD, two models were fitted: an unadjusted model including only race/ethnicity, and a multivariable model adjusting for a comprehensive set of demographic, clinical, environmental, and behavioral covariates guided by the integrated health risk management (IHRM) framework. Adjusted analyses included age (modeled continuously), sex, body mass index (BMI), and primary cause of ESRD, including diabetes, hypertension, glomerulonephritis, cystic kidney disease, and other or unknown etiologies. Additional covariates included employment status, insurance coverage, smoking status, alcohol and drug dependence, and functional status indicators such as assistance with activities of daily living and inability to ambulate. Comorbidities relevant to ESRD progression and treatment suitability were incorporated using categorical coding based on USRDS definitions. Missing data were handled using complete‐case analysis. Although the proportion of missingness across included covariates was low, we recognize that missingness in administrative datasets such as the USRDS may be related to health care utilization, clinical severity, or documentation practices rather than occurring completely at random. Consequently, complete‐case analysis may introduce selection bias, and the possibility of residual bias related to missing data cannot be excluded.

To further explore potential mechanisms underlying observed racial and ethnic disparities, additional exploratory analyses were conducted using variables available in the USRDS Medical Evidence Form (CMS‐2728). First, interaction analyses were performed to evaluate whether employment status and insurance coverage modified the association between race/ethnicity and dialysis modality initiation. Separate Poisson regression models with robust standard errors were fitted for HHD versus ICHD and PD versus ICHD, incorporating interaction terms between race/ethnicity and employment status and between race/ethnicity and insurance category. Global Wald tests were used to assess the statistical significance of interaction terms. Second, sensitivity analyses were conducted to evaluate the influence of pre‐ESKD nephrology care on the observed associations. Duration of nephrology care prior to ESRD onset was categorized as no nephrology care, < 6 months, 6–12 months, or > 12 months and added to the multivariable models. Adjusted prevalence ratios from these models were compared with the primary adjusted estimates to determine whether differences in access to specialty nephrology care attenuated the observed racial and ethnic disparities in dialysis modality initiation.

All statistical analyses were conducted using Stata version 17.0 (StataCorp LLC). Statistical significance was determined using two‐sided p‐values with a threshold of < 0.05.

### 2.6 Ethical Considerations

This study used de‐identified secondary data from the United States Renal Data System (USRDS). Data access and use were conducted under a formal data use agreement with the USRDS, and all applicable data security and confidentiality requirements were followed.

## 3 Results

### 3.1 Biological Determinants

Between 2014 and 2020, a total of 3,485,846 adult patients initiated dialysis therapy for end‐stage renal disease in the United States. Of these, 2,885,867 (82.8%) began treatment with in‐center hemodialysis (ICHD), 60,525 (1.7%) with home hemodialysis (HHD), and 539,454 (15.5%) with peritoneal dialysis (PD). The overall cohort was predominantly male (56.6%), with a median age of 64 years among patients initiating ICHD and 59 years among those initiating either HHD or PD (Table 1).

**TABLE 1:** Biological determinants of incident ESRD patients by initial dialysis modality.

Characteristic	ICHD (*n* = 2,885,867)	%	HHD (*n* = 60,525)	%	PD (*n* = 539,454)	%	Total (*N* = 3,485,846)	%
**Gender**								
Male	1702,878	59.04	37,224	61.52	231,942	42.97	1,972,044	56.56
Female	1,305,688	45.23	24,089	39.81	184,025	34.11	1,513,802	43.44
**Median age (IQR)**	64 (54–74)	—	59 (44–69)	—	59 (44–69)	—	64 (54–73)	—
**Race/ethnicity**								
White (NH)	1,431,566	49.61	34,685	57.31	238,372	44.17	1,704,623	48.92
Black/African American (NH)	954,890	33.09	19,162	31.68	97,129	18.00	1,071,181	30.73
American Indian/Alaska Native (NH)	34,296	1.19	321	0.53	3892	0.72	38,509	1.10
Asian (NH)	102,958	3.57	1544	2.55	19,914	3.69	124,416	3.57
Native Hawaiian/Pacific Islander (NH)	32,475	1.13	361	0.60	3919	0.73	36,755	1.05
Other (NH)	5452	0.19	125	0.21	823	0.15	6400	0.18
Hispanic or Latino (any race)	446,892	15.48	5115	8.45	51,917	9.62	503,924	14.46
**Anthropometrics**								
Median height, cm (IQR)	167.6 (160.1–176)	—	170.2 (162.6–178)	—	170 (162–177.8)	—	168 (160–176)	—
Median weight, kg (IQR)	78 (65–95)	—	83 (68.1–103)	—	80 (68–95.3)	—	76 (66–96)	—
Median BMI, kg/m^2^ (IQR)	27.5 (23.5–33.1)	—	28.6 (24.1–34.9)	—	27.8 (24.1–32.7)	—	27.7 (23.6–33.3)	—
**Primary cause of ESRD**								
Diabetes	1,350,288	46.81	23,976	39.62	222,049	41.17	1,596,313	45.80
Hypertension	820,956	28.45	16,549	27.34	136,190	25.24	973,695	27.95
Glomerulonephritis	266,026	9.22	8456	13.97	93,193	17.28	367,675	10.55
Cystic kidney disease	57,526	1.99	2291	3.78	25,138	4.66	84,955	2.44
**Comorbidities**								
Diabetes	1,586,288	55.00	24,976	41.28	319,049	59.14	1,930,313	55.40
Hypertension	2,310,956	80.10	48,549	80.22	378,190	70.09	2,737,695	78.60
AIDS	1548	0.05	11	0.02	143	0.03	1702	0.05
Cancer	121,866	4.22	1400	2.31	13,413	2.49	136,679	3.92
COPD	160,779	5.57	1840	3.04	11,812	2.19	174,431	5.01
Glomerulonephritis	288,026	9.98	9486	15.68	94,173	17.46	391,685	11.24
Cystic kidney	57,726	2.00	3241	5.36	25,138	4.66	86,105	2.47

*Note:* Height, weight, and BMI contained minimal missingness (< 0.5%). Remaining missing values were retained and included in the denominator; therefore, percentages may not sum to 100%. BMI was calculated from height and weight at dialysis initiation. Primary cause of ESRD and comorbidities were derived from CMS Form 2728.

The distribution of the newly constructed race/ethnicity variable differed markedly across dialysis modalities. Among patients initiating ICHD, White non‐Hispanic patients accounted for 49.6% (1,431,566) of initiations, whereas Black non‐Hispanic patients represented 33.1% (954,890). In contrast, PD users were more frequently White non‐Hispanic (44.2%; 238,372) and less often Black non‐Hispanic (18.0%; 97,129). A similar pattern was evident in HHD, where White non‐Hispanic patients comprised 57.3% (34,685) of initiators, compared with 31.7% (19,162) who were Black non‐Hispanic.

Patients identifying as Hispanic or Latino (any race) constituted 15.4% (446,892) of ICHD initiators but represented only 9.7% (51,917) of PD initiators and 8.5% (5115) of HHD initiators, indicating consistent underrepresentation in both home‐based modalities (Table 1).

### 3.2 Environmental Determinants

Employment status differed markedly across dialysis modalities. Patients initiating HHD or PD were more likely to be employed, particularly full‐time, compared with those starting ICHD. Full‐time employment was highest among PD initiators (10.1%) and HHD initiators (4.4%), compared with only 4.7% among ICHD patients. In contrast, unemployment was most prevalent among ICHD patients (14.7%), followed by PD (9.1%) and HHD (5.6%). Disability‐related retirement was also more common among ICHD users (13.5%) than among HHD (6.0%) or PD (8.2%) patients, reflecting a greater burden of socioeconomic vulnerability in the in‐center population.

Patterns of health care coverage showed similar gradients. Medicare was the dominant insurer for ICHD patients (35.9%), whereas its prevalence was markedly lower among HHD (16.8%) and PD (21.9%) initiators. Conversely, private or “other” insurance types were more frequently represented in both HHD (6.7%) and PD (9.9%) groups compared with ICHD (13.8%, though still high due to the size of the ICHD population). Medicaid coverage was highest among ICHD initiators (17.2%), consistent with greater economic disadvantage. Lack of insurance was relatively uncommon overall but most prevalent among PD initiators (3.3%) and least common among HHD patients (1.2%) (Table 2).

**TABLE 2:** Environmental determinants of incident ESRD patients by initial dialysis modality.

Characteristic	ICHD (*n* = 2,885,867)	%	HHD (*n* = 60,525)	%	PD (*n* = 539,454)	%	Total (*N* = 3,485,846)	%
**Employment status**								
Unemployed	423,570	14.68	3373	5.57	49,037	9.09	475,570	13.65
Full‐time employed	134,384	4.66	2666	4.40	54,288	10.06	191,338	5.49
Part‐time employed	35,214	1.22	462	0.76	10,369	1.92	46,045	1.32
Homemaker	57,943	2.01	374	0.62	8363	1.55	66,680	1.91
Retired (age‐related)	693,545	24.04	6759	11.17	74,099	13.74	774,403	22.22
Retired (disability)	389,578	13.50	3603	5.95	44,269	8.21	437,450	12.55
**Health care coverage**								
Medicare	1,034,704	35.85	10,193	16.84	117,880	21.86	1,162,777	33.36
Medicaid	495,202	17.16	3781	6.25	41,197	7.63	540,180	15.50
Department of Veterans Affairs (DVA)	36,218	1.26	238	0.39	3630	0.67	40,086	1.15
Other insurance	397,456	13.78	4078	6.74	53,319	9.88	454,853	13.05
No medical insurance	129,237	4.48	726	1.20	17,807	3.30	147,770	4.24
**Pre‐ESKD nephrology care**								
No nephrology care	599,933	17.70	1307	11.36	22,701	5.87	623,941	16.47
< 6 months	290,647	8.57	816	7.09	38,957	10.07	330,420	8.72
6–12 months	443,884	13.09	1174	10.20	66,503	17.19	511,561	13.50
> 12 months	628,221	18.53	1929	16.76	119,412	30.87	749,562	19.78

*Note:* Missing or unclassified values were included in denominators unless otherwise noted. Employment categories reflect patient‐reported or administrative documentation on CMS Form 2728. Insurance categories correspond to primary payer at ESRD onset. Pre‐ESKD nephrology care categories were derived from CMS Medical Evidence Form (CMS‐2728) data and represent the nephrology care prior to ESRD onset. Because denominators differ by modality, comparisons should be made within rows rather than across columns.

### 3.3 Behavioral Determinants

Behavioral risk factors, including alcohol dependence, drug dependence, and active smoking, were consistently more common among patients initiating ICHD compared with those beginning HHD or PD. Alcohol dependence was reported in 0.9% of ICHD patients, compared with 0.4% of HHD patients and 0.3% of PD patients. Drug dependence followed a similar pattern, occurring in 0.8% of those starting ICHD, 0.3% initiating HHD, and 0.2% initiating PD. Smoking prevalence was highest among ICHD initiators (3.8%), lower among PD initiators (2.9%), and lowest in HHD patients (1.3%).

Functional status indicators also differed markedly by modality. Dependence in activities of daily living (ADLs) was most frequent among ICHD patients (6.1%), compared with 5.2% in HHD and only 1.6% in PD, indicating a substantially higher degree of functional independence among home‐therapy users. Similarly, inability to ambulate was far more common in ICHD patients (3.8%) relative to those initiating HHD (4.1%) or PD (0.6%). PD initiators demonstrated the greatest functional independence, while HHD users showed intermediate functional status but still markedly better mobility than the ICHD population (Table 3).

**TABLE 3:** Behavioral determinants of incident ESRD patients by initial dialysis modality.

Characteristic	ICHD (*n* = 2,885,867)	%	HHD (*n* = 60,525)	%	PD (*n* = 539,454)	%	Total (*N* = 3,485,846)	%
**Alcohol dependence**								
Yes	26,949	0.93	214	0.35	1386	0.26	28,549	0.82
No	1,729,533	59.95	17,556	29.03	248,236	46.03	1,995,325	57.23
**Drug dependence**								
Yes	23,904	0.83	166	0.27	1119	0.21	25,189	0.72
No	1,732,578	60.03	17,604	29.10	248,503	46.08	1,998,685	57.34
**Smoking**								
Yes	110,844	3.84	779	1.29	15,376	2.85	126,999	3.64
No	1,645,638	56.99	16,991	28.08	234,246	43.42	1,896,875	54.43
**Assistance with daily living**								
Yes	175,542	6.09	3128	5.17	84,420	15.65	263,090	7.55
No	1,580,940	54.81	14,642	24.19	241,202	44.71	1,836,784	52.70
**Unable to ambulate**								
Yes	110,280	3.82	2481	4.10	3343	0.62	116,104	3.33
No	1,646,202	57.04	15,289	25.28	246,279	45.66	1,907,770	54.73

*Note:* Missing values for smoking, drug/alcohol dependence, assistance with activities of daily living, and ambulation status were included in the denominators of each modality group. Alcohol and drug dependence, smoking status, assistance with ADLs, and inability to ambulate were derived from CMS Form 2728 checkboxes. Behavioral variables were coded as binary (Yes/No), consistent with USRDS conventions.

### 3.4 Racial and Ethnic Disparities in Modality Initiation

Multivariable Poisson regression with robust variance was used to estimate adjusted incidence rate ratios (IRRs) for initiating HHD or PD compared with ICHD, incorporating biological, environmental, and behavioral covariates aligned with the IHRM framework. The results demonstrated persistent and statistically significant racial and ethnic differences in the use of home‐based dialysis modalities (Table 4).

**TABLE 4:** Unadjusted and adjusted prevalence ratios (PR) for initiating home hemodialysis (HHD) or peritoneal dialysis (PD) versus in‐center hemodialysis (ICHD), by race/ethnicity (2014–2020).

Predictor	HHD vs. ICHD		PD vs. ICHD	
Race/ethnicity^a^	Crude PR (95% CI)^b^	Adjusted PR (95% CI)^c^	Crude PR (95% CI)^b^	Adjusted PR (95% CI)^c^
White (non‐Hispanic)	1.00 (ref)	1.00 (ref)	1.00 (ref)	1.00 (ref)
Black (non‐Hispanic)	0.81 (0.80–0.83)	0.69 (0.67–0.72)	0.64 (0.63–0.64)	0.55 (0.54–0.56)
American Indian (non‐Hispanic)	0.38 (0.35–0.43)	0.35 (0.28–0.44)	0.71 (0.69–0.73)	0.63 (0.60–0.65)
Asian (non‐Hispanic)	0.63 (0.60–0.67)	0.67 (0.61–0.74)	1.14 (1.12–1.15)	1.08 (1.06–1.09)
Native Hawaiian (non‐Hispanic)	0.46 (0.42–0.51)	0.39 (0.31–0.48)	0.76 (0.74–0.78)	0.68 (0.66–0.71)
Other/unknown (non‐Hispanic)^d^	0.96 (0.80–1.14)	1.19 (0.90–1.57)	0.93 (0.87–0.99)	0.82 (0.75–0.89)
Hispanic (any race)	0.48 (0.47–0.49)	0.42 (0.40–0.45)	0.73 (0.72–0.74)	0.64 (0.64–0.65)

^a^ Race/ethnicity modeled as a combined variable. Individuals reporting Hispanic ethnicity were classified as “Hispanic” regardless of race; all others were categorized into non‐Hispanic racial groups using USRDS definitions.

^b^ Crude prevalence ratios (PR) estimated using Poisson regression with robust variance and race/ethnicity as the only predictor (White non‐Hispanic as reference).

^c^ Adjusted PRs estimated from Poisson regression models with robust variance including race/ethnicity, age, sex, BMI, and primary cause of ESRD.

^d^ “Other/Unknown” includes non‐Hispanic individuals classified as Other race and those with unknown race; estimates should be interpreted with caution due to small cell sizes.

For HHD, Black patients had a 31% lower incidence rate of initiation than White patients (IRR: 0.69, 95% CI 0.67–0.72). Hispanic or Latino patients showed an even greater disparity, with a 58% lower incidence rate of HHD initiation compared with non‐Hispanic White patients (IRR: 0.42, 95% CI 0.40–0.45). American Indian/Alaska Native patients (IRR: 0.35, 95% CI 0.28–0.44), Asian patients (IRR: 0.67, 95% CI 0.61–0.74), and Native Hawaiian or Pacific Islander patients (IRR: 0.39, 95% CI 0.31–0.48) also had significantly lower HHD initiation rates. These findings indicate that home hemodialysis initiation was consistently less common across nearly all minority groups, even after covariate adjustment.

The pattern was similar for PD initiation, although with important differences. After adjustment, Black patients had a 45% lower incidence rate of starting PD compared with White patients (IRR: 0.55, 95% CI: 0.54–0.56), while Hispanic or Latino patients had a 36% lower PD initiation rate (IRR: 0.64, 95% CI: 0.64–0.65). American Indian/Alaska Native and Native Hawaiian or Pacific Islander patients also exhibited substantially lower PD initiation rates (IRRs: 0.63 and 0.68, respectively). In contrast, Asian patients were more likely than White patients to initiate PD, with an IRR of 1.08 (95% CI: 1.06–1.09), indicating an 8% higher adjusted incidence rate.

As exploratory assessments of potential mechanisms underlying observed disparities, additional analyses were conducted using measures of socioeconomic resources, healthcare access, and pre‐ESKD specialty care. Interaction analyses demonstrated significant effect modification by both employment status and insurance coverage for HHD and PD initiation (all *p* < 0.001; Table S2). However, the overall pattern of lower home dialysis utilization among Black and Hispanic patients remained evident across categories. In addition, sensitivity analyses incorporating pre‐ESKD nephrology care showed that longer nephrology follow‐up prior to ESRD onset was strongly associated with initiation of home dialysis modalities. Adjustment for pre‐ESKD nephrology care modestly attenuated the associations observed among Black and Hispanic patients, particularly for PD initiation, but substantial disparities persisted after adjustment (Table S3).

## 4 Discussion and Conclusions

This study analyzed nationally representative data from 2014 to 2020 to evaluate potential racial and ethnic disparities in the initial selection of renal replacement therapy (RRT) among adults with incident end‐stage renal disease (ESRD) in the United States. The patterns observed show persistent differences in access to home dialysis modalities, home hemodialysis (HHD) and peritoneal dialysis (PD), compared with in‐center hemodialysis (ICHD). These disparities were particularly pronounced among Black and Hispanic or Latino populations. Such differences remained even after adjusting for certain related factors: compared with White patients, Black individuals had a 31% lower adjusted incidence rate of initiating HHD and a 45% lower rate of initiating PD. Hispanic or Latino patients showed even greater disparities: their adjusted incidence rate of initiating HHD was 58% lower and that of initiating PD was 36% lower than that of non‐Hispanic White patients. On the other hand, Asian patients had an 8% higher incidence rate of initiating PD compared with White patients. Other minority groups, including American Indian/Alaska Native and Native Hawaiian/Pacific Islander patients, showed consistently lower adjusted use of both home modalities.

Taken together, these results reinforce previous research suggesting that factors such as structural barriers, variability in referral to nephrology, insufficient education prior to dialysis, and provider‐level influences may contribute to lower access to home RRT among minority populations [3, 5]. Additional exploratory analyses incorporating pre‐ESKD nephrology care demonstrated that longer nephrology follow‐up prior to ESRD onset was strongly associated with initiation of home dialysis modalities. Furthermore, adjustment for pre‐ESKD nephrology care modestly attenuated the observed associations among Black and Hispanic patients, particularly for PD initiation, suggesting that differences in access to specialty nephrology care may partially contribute to the observed disparities. However, substantial disparities remained after adjustment, indicating that variation in measured nephrology care alone does not fully explain the observed differences. The persistence of disparities even after adjustments suggests the presence of unmeasured contextual factors, including the home environment, caregiver availability, health literacy, language concordance, and regional differences in modality expertise, which continue to influence modality choice at the onset of ESRD [10].

The application of the Integrated Health Risk Management (IHRM) framework provided a structured approach for interpreting observed differences in dialysis modality initiation by considering the combined influence of biological, behavioral, and environmental determinants. Although disparities persisted after adjustment for measured clinical and sociodemographic factors, the observational nature of the study and the absence of detailed measures of predialysis education, referral patterns, provider practices, social support, and other contextual factors limit our ability to identify the specific mechanisms underlying these differences [6, 11]. Notably, additional analyses incorporating duration of pre‐ESKD nephrology care, an environmental determinant reflecting access to specialty care, demonstrated only partial attenuation of the observed disparities. These findings suggest that although access to nephrology care is an important contributor, additional clinical, social, structural, and health system factors likely influence modality selection. Within this context, the IHRM framework offers a useful structure for considering multiple domains of influence and identifying potential areas for future investigation and intervention.

Exploratory analyses further demonstrated that employment status and insurance coverage modified the magnitude of racial and ethnic disparities in both HHD and PD initiation. These findings suggest that socioeconomic resources and healthcare access may influence modality selection. However, the persistence of disparities across employment and insurance strata indicates that these factors do not fully explain the observed differences and that additional clinical, structural, and systemic influences likely contribute to variation in home dialysis utilization.

Previous studies suggest that structured predialysis education programs can increase awareness of dialysis options and may improve uptake of home‐based therapies in selected populations [12]. Multidisciplinary chronic kidney disease care models have also been associated with improved modality preparedness and fewer unplanned dialysis starts [13]. Similarly, standardized decision aids, early referral to nephrology teams experienced in home therapies [14], and culturally tailored educational approaches may help address informational and communication barriers among historically underserved populations [15]. Facility‐level strategies, including PD‐first protocols, expansion of home dialysis training capacity, and telehealth support, have also been associated with increased home dialysis utilization in some settings [3].

However, the evidence that these interventions consistently reduce racial and ethnic disparities at the population level remains limited and mixed. While targeted programs may improve access and preparedness in specific clinical contexts, sustained reductions in disparities have been variable across health systems and geographic settings. Therefore, these approaches should be interpreted as potential areas for intervention rather than proven solutions for eliminating racial and ethnic differences in home dialysis initiation. Within this context, the IHRM framework provides a useful structure for considering how multiple biological, behavioral, and environmental factors may be associated with modality selection and for identifying priorities for future research and intervention.

An important consideration highlighted in previous research and relevant to our findings is the role of quality‐of‐life (QOL) preferences and shared decision‐making between patient and family [16]. Studies have consistently shown that PD can offer greater autonomy, flexibility, and lifestyle compatibility compared with in‐center hemodialysis [17]. However, home therapies require adequate functional capacity, an appropriate home environment, and reliable caregiver support and therefore may be less feasible among patients with limited social resources, high comorbidity burden, frailty, or significant symptoms [18]. Since modality choice often depends on a collaborative process between the patient, family, and the nephrology team, shared decision‐making constitutes the recommended standard to ensure alignment between clinical suitability and patient values [19]. These considerations are especially relevant in the context of racial and ethnic differences, as previous studies have shown that the availability of family support, cultural expectations around caregiving, work constraints, and housing conditions vary systematically between groups and can influence the feasibility of or access to home therapies [3]. Consequently, some of the observed differences in PD and HHD initiation may reflect differences in social support structures and environmental resources rather than inequity alone.

It is also important to mention that differences in modality initiation between racial and ethnic groups should not automatically be assumed to represent inequity, nor should it be presumed that treatment choice must be identical across all sociodemographic groups. The suitability of home dialysis depends on the combination of clinical, functional, environmental, and social factors, which can vary significantly between populations [20]. In some contexts, demographic patterns such as older age, greater medical complexity, or more limited home resources may legitimately reduce the feasibility of PD or HHD [21]. In turn, certain groups may have cultural or family support structures that facilitate the adoption of home therapies. These factors underscore that not all variation is inherently inappropriate; rather, the observed differences should be understood within a broader clinical and social context. At the same time, the persistence of disparities after adjustment for measured variables suggests that unmeasured contextual influences and system‐level factors may still contribute to differential access.

Additionally, the interpretation of our findings must also recognize that differences in modality initiation between racial and ethnic groups do not necessarily imply inappropriate care, implicit bias, or racism. Part of the variation may reflect complex social, cultural, and medical circumstances that RRT professionals cannot control or measure, but that may legitimately influence modality suitability and patient preference. Factors such as multigenerational caregiving patterns, cultural norms around family participation in care, housing constraints, frailty, and comorbidity burden can shape decisions in ways that are appropriate and consistent with patient values [22]. In this context, divergent modality patterns may, at times, represent a rational alignment between patient needs, household resources, and the flexibility of the U.S. RRT system rather than inequity.

Furthermore, there are different schools of thought on how disparities should be interpreted. While some research emphasizes the potential role of structural barriers in shaping access to care [23], other perspectives warn that disparities alone do not constitute evidence of systemic bias and caution against over‐attributing differences to systemic racism in the absence of comprehensive contextual data [24]. These viewpoints underscore that modality initiation is influenced by a broad mix of measurable factors, unmeasured contextual influences, patient preferences, resource constraints, and appropriate clinical decision‐making. Selection bias in how modalities are offered may also shape the observed patterns. PD and HHD are frequently directed toward patients with adequate family support, fewer comorbidities, higher educational attainment, and stable home environments [25], whereas patients with advanced frailty, poorly controlled anemia, inadequate dialysis adequacy, or complex clinical profiles may be preferentially directed to ICHD [26]. Because our dataset lacked granular measures of social support, caregiver availability, home conditions, or educational level, we were unable to directly adjust for these influential factors.

International evidence also supports the importance of provider influence in dialysis modality selection. In Japan, Ikeda et al. demonstrated that the availability and involvement of nephrologists significantly shaped whether patients initiated PD [27]. Another provider‐level factor is variation in nephrologists' exposure to, training in, and confidence with home dialysis; clinicians with limited training may be less likely to recommend home modalities [28]. Facilities serving minority or socioeconomically disadvantaged patients may also have fewer resources for home therapies, further interacting with broader social inequities. Interestingly, Asian patients were more likely than White patients to initiate PD, consistent with prior studies reporting higher PD utilization in some Asian populations [3, 29, 30, 31]. Several explanations have been proposed in the literature, including greater familiarity with home‐based therapies, cultural preferences regarding family involvement in care, and differences in health system practices and modality education. However, the mechanisms underlying these patterns remain incompletely understood, and our study was not designed to evaluate the specific social, cultural, or clinical factors contributing to higher PD uptake among Asian patients. These findings underscore that differences between groups cannot always be attributed solely to inequity. In some circumstances, variation in dialysis modality selection may appropriately reflect informed patient preferences, shared decision‐making between patients and clinicians, individualized clinical judgment, and the availability of caregiver support. Respecting patient values and treatment preferences is a fundamental component of patient‐centered kidney care. Therefore, while reducing unwarranted barriers to home dialysis remains essential, efforts to improve equity should also preserve patient autonomy and individualized treatment decision‐making.

Several limitations must be recognized when interpreting these findings. First, the analysis was based on secondary data from the United States Renal Data System (USRDS), which, although comprehensive, may be subject to inaccuracies or omissions in clinical and demographic reporting. For example, race and ethnicity are self‐reported or administratively assigned and may not fully capture the cultural diversity within these broad categories. Second, an additional limitation relates to the requirement that patients remain on the same dialysis modality for at least 60 days following initiation, consistent with USRDS conventions for modality assignment. Although this approach improves the reliability of modality classification and reduces misclassification arising from short‐term treatment transitions, it may also introduce selection bias by excluding patients who experience early modality failure or rapid switching between therapies. If these events occur disproportionately among certain racial or ethnic groups because of differences in clinical complexity, social support, access to training, or other unmeasured factors, the observed disparities may underestimate the true magnitude of differences in access to and successful initiation of home‐based dialysis modalities.

Third, the USRDS dataset lacks detailed systemic, structural, and social determinants, including measures of key social factors that influence modality choice, such as health literacy, language proficiency, caregiver support, educational attainment, home environment, and transportation access. These unmeasured factors may partially explain the observed disparities and limit the ability to fully adjust for contextual influences. In addition, missing data were addressed using complete‐case analysis. Although the overall proportion of missingness was low, missingness in administrative datasets may be associated with differences in health care utilization, disease severity, or documentation practices rather than occurring completely at random. Therefore, some degree of selection bias related to missing data cannot be ruled out. This analysis also lacked detailed clinical parameters such as anemia severity, dialysis adequacy metrics, inflammatory status, and comprehensive measures of predialysis education and care quality. Although duration of pre‐ESKD nephrology care was available and incorporated into supplementary analyses, the dataset did not capture the content, quality, timing of referral, or effectiveness of nephrology care and education, which may influence suitability for PD or HHD and contribute to modality selection. Although multivariable regression models were used to adjust for a range of covariates, unobserved confounding cannot be ruled out. In addition, provider‐level factors such as nephrologist preferences and dialysis facility characteristics were not captured in the dataset. This analysis did not examine longitudinal outcomes such as modality switching, hospitalization, or mortality, which could provide further insight into the implications of initial modality disparities over time. In addition, although the study included incident ESRD cases from 2014 through 2020, temporal changes in home dialysis utilization and racial/ethnic disparities were not evaluated. Future research examining trends over time may help determine whether disparities have narrowed, persisted, or widened in response to evolving clinical practices and national initiatives promoting home dialysis.

Furthermore, although the study adjusted for age, sex, body mass index, and primary cause of ESRD, these adjustments were intended to account for potential confounding and do not address the separate question of whether the magnitude of racial and ethnic disparities varies across patient subgroups. The present analysis focused on overall racial and ethnic differences in modality initiation and did not comprehensively evaluate effect modification according to age, comorbidity burden, geographic region, or other patient characteristics. Future studies examining subgroup‐specific disparities may help identify populations at particularly high risk of inequitable access to home dialysis therapies. Although our findings may inform policy and practice, the observational design necessarily limits causal inference, and we cannot conclude that specific interventions will directly reduce racial and ethnic disparities in home dialysis initiation. While several educational, clinical, and system‐level interventions have shown promise in improving home dialysis utilization in selected settings, evidence regarding their effectiveness in achieving sustained reductions in racial and ethnic disparities at the population level remains limited and inconsistent. This likely reflects the complex interplay of clinical, social, cultural, and health system factors that influence modality selection.

In conclusion, this study highlights persistent racial and ethnic disparities in the initiation of renal replacement therapy among patients with incident ESRD in the United States from 2014 to 2020. Black and Hispanic or Latino patients were significantly less likely to initiate home dialysis modalities compared with their White and non‐Hispanic counterparts, even after adjustment for multiple covariates. Although structural and system‐level factors likely contribute, patient‐level clinical considerations, cultural norms, social support availability, provider practice patterns, and informed patient preferences also play important roles. Consequently, efforts to reduce disparities should focus on eliminating unwarranted barriers to home dialysis while preserving shared decision‐making, patient autonomy, and individualized treatment selection. Using the Integrated Health Risk Management (IHRM) framework as an organizing structure, this study highlights that disparities in dialysis modality initiation are associated with a complex set of biological, behavioral, environmental, and potentially structural factors that may influence access to home‐based therapies.

## Funding

The authors have nothing to report.

## Ethics Statement

This study was exempted from ethical review by the University of Central Florida Institutional Review on 9/24/2021 under IRB ID STUDY00003457 and FWA00000351 IRB00001138, and IRB00012110.

## Conflicts of Interest

The authors declare no conflicts of interest.

## Supporting information


**Table S1:** Variable Definitions, Coding, and Rationale for Inclusion.
**Table S2:** Exploratory interaction analyses evaluating employment status and insurance coverage as proxies for socioeconomic resources and healthcare access.
**Table S3:** Adjusted Prevalence Ratios (PR) for Initiating Home Hemodialysis (HHD) or Peritoneal Dialysis (PD) vs. In‐Center Hemodialysis (ICHD), After Additional Adjustment for Pre‐ESKD Nephrology Care (2014–2020).

## Data Availability

The data that support the findings of this study are available on request from the corresponding author. The data are not publicly available due to privacy or ethical restrictions.
