# Supplementary material for: Racial and Ethnic Disparities in Access to Home‐Based Renal Replacement Therapies Among Incident End‐Stage Renal Disease Patients in the United States
Source: Semin Dial. 2026 Oct 4;39(5):266–78. doi: 10.1111/sdi.70050 (PMC13716842; doi:10.1111/sdi.70050)
Supplement: Supplementary file 1 [file SDI-39-266-s001.docx]

**Supplementary materials**

**Supplementary Table S1. Variable Definitions, Coding, and Rationale for Inclusion**

| **Variable** | **Definition & Coding** | **Source** | **Rationale for Inclusion** |
| --- | --- | --- | --- |
| **Race/Ethnicity** | Classified as White, Black/African American, Asian, American Indian/Alaska Native, Native Hawaiian/Pacific Islander, Other, Hispanic or Latino | USRDS administrative file | Primary exposure; prior evidence of modality disparities |
| **Age** | Continuous years at ESRD onset | CMS-2728 | Strong predictor of functional status, home dialysis feasibility |
| **Sex** | Male or Female | CMS-2728 | Influences comorbidities, caregiving patterns, modality uptake |
| **BMI** | Continuous (kg/m²) calculated from height and weight | CMS-2728 | Extreme BMI affects PD catheter placement and HHD feasibility |
| **Primary Cause of ESRD** | Diabetes, hypertension, glomerulonephritis, cystic kidney disease, other | CMS-2728 | Underlying disease affects CKD progression, predialysis care |
| **Comorbidities** | Binary (yes/no): diabetes, hypertension, COPD, cancer, AIDS, glomerulonephritis, cystic kidney | CMS-2728 | Medical complexity affects modality suitability and stability |
| **Employment Status** | Full-time, part-time, unemployed, homemaker, retired-age, retired-disability | CMS-2728 | Proxy for socioeconomic status, social support, time constraints |
| **Insurance Type** | Medicare, Medicaid, DVA, other, uninsured | CMS-2728 | Influences access to nephrology care and modality education |
| **Smoking** | Yes/No | CMS-2728 | Behavioral risk factor influencing clinical recommendations |
| **Alcohol Dependence** | Yes/No | CMS-2728 | Affects self-management capacity and modality suitability |
| **Drug Dependence** | Yes/No | CMS-2728 | May influence home therapy safety and adherence |
| **Assistance with ADLs** | Yes/No | CMS-2728 | Indicator of functional limitations affecting home therapy |
| **Inability to Ambulate** | Yes/No | CMS-2728 | Captures severe functional impairment impacting modality choice |

Supplementary Table S2. Exploratory interaction analyses evaluating employment status and insurance coverage as proxies for socioeconomic resources and healthcare access

| Outcome | Interaction Term | χ² | df | p-value |
| --- | --- | --- | --- | --- |
| HHD vs ICHD | Race/Ethnicity × Employment Status | 18,634.98 | 56 | <0.001 |
| HHD vs ICHD | Race/Ethnicity × Insurance Category | 2,641.39 | 27 | <0.001 |
| PD vs ICHD | Race/Ethnicity × Employment Status | 1,652.70 | 56 | <0.001 |
| PD vs ICHD | Race/Ethnicity × Insurance Category | 619.47 | 27 | <0.001 |

Supplementary Table S3. Adjusted Prevalence Ratios (PR) for Initiating Home Hemodialysis (HHD) or Peritoneal Dialysis (PD) vs In-Center Hemodialysis (ICHD), After Additional Adjustment for Pre-ESKD Nephrology Care (2014–2020)

| Predictor | HHD vs ICHD | PD vs ICHD |
| --- | --- | --- |
| Race/Ethnicity* | Adjusted PR (95% CI)† | Adjusted PR (95% CI)† |
| White (non-Hispanic) | 1.00 (ref) | 1.00 (ref) |
| Black (non-Hispanic) | 0.68 (0.66–0.70) | 0.61 (0.61–0.62) |
| American Indian (non-Hispanic) | 0.40 (0.35–0.45) | 0.68 (0.65–0.70) |
| Asian (non-Hispanic) | 0.62 (0.58–0.66) | 1.05 (1.03–1.06) |
| Native Hawaiian (non-Hispanic) | 0.45 (0.40–0.51) | 0.71 (0.68–0.73) |
| Other (non-Hispanic) | 0.79 (0.65–0.97) | 0.82 (0.77–0.88) |
| Hispanic (any race) | 0.44 (0.42–0.45) | 0.71 (0.70–0.71) |

*Race/ethnicity modeled as a combined variable. Individuals reporting Hispanic ethnicity were classified as “Hispanic” regardless of race; all others were categorized into non-Hispanic racial groups using USRDS definitions.

†Adjusted prevalence ratios (PRs) estimated from Poisson regression models with robust variance including race/ethnicity, age, sex, BMI, primary cause of ESRD, and pre-ESKD nephrology care. Nephrology care was categorized as no nephrology care, <6 months, 6–12 months, and >12 months prior to ESKD onset based on CMS Medical Evidence Form (CMS-2728) data.
